# Renal metastasis of ovarian granulosa cell tumor

**DOI:** 10.1002/iju5.12433

**Published:** 2022-03-21

**Authors:** Kyo Togashi, Tohru Yoneyama, Mihoko Sutoh Yoneyama, Hayato Yamamoto, Shingo Hatakeyama, Takahiro Yoneyama, Yasuhiro Hashimoto, Masayuki Futagami, Chikara Ohyama

**Affiliations:** ^1^ Department of Urology Hirosaki University Graduate School of Medicine Hirosaki Japan; ^2^ Department of Glycotechnology Center for Advanced Medical Research Hirosaki University Graduate School of Medicine Hirosaki Japan; ^3^ Department of Cancer Immunology and Cell Biology Oyokyo Kidney Research Institute Hirosaki Japan; ^4^ Department of Obstetrics and Gynecology Hirosaki University Graduate School of Medicine Hirosaki Japan

**Keywords:** adult granulosa cell tumor, c134w mutation, FOXL2, renal metastasis

## Abstract

**Introduction:**

We would like to present a rare case of metastatic renal tumor.

**Case presentation:**

A 60‐year‐old woman presented to our department with a left renal tumor. She underwent a total hysterectomy and right adnexal resection for a stage IA ovarian granulosa cell tumor approximately 15 years ago, followed by left adnexal resection and postoperative chemotherapy with gemcitabine and paclitaxel 6 years ago. She received six courses of gemcitabine and carboplatin to treat a stage IC clear cell adenocarcinoma of the ovary.

The patient was diagnosed with the left renal tumor and underwent a laparoscopic left nephrectomy. Immunostaining was positive for α‐inhibin and SF‐1 and showed FOXL2 402C→G (C134W) mutation. Finally, the patient was diagnosed with renal metastasis of a granulosa cell tumor.

**Conclusion:**

To our knowledge, this is a very rare case of renal metastasis of a granulosa cell tumor with the FOXL2 mutation in an adult.

**Abbreviations & Acronyms**
AGCT: adult granulosa cell tumor
cDNA: complementary deoxyribonucleic acid
CT: computed tomography
ddPCR: droplet digital polymerase chain reaction
FFPE: formalin‐fixed paraffin‐embedded
GCT: granulosa cell tumors
gDNA: genomic deoxyribonucleic acid
MRI: magnetic resonance imaging
PCR: polymerase chain reaction
SRY: sex‐determining Region Y

**Keynote message**

We present a rare case report of a patient with a late adult ovarian granulosa cell tumor recurrence in the left kidney.

## Introduction

Ovarian GCTs originate from follicular granulosa cells and are reported to account for <5% of all ovarian malignancies. When they extend outside the ovary, they remain in the pelvis; hence, distant metastasis is rare. Herein, we describe a rare case of renal metastasis of AGCT 15 years after the primary treatment.

## Case presentation

The patient was a 60‐year‐old woman. In 2002, she underwent surgery for a right ovarian tumor and was diagnosed with a stage IA AGCT. In 2011, she developed clear cell ovarian cancer in her left ovary and underwent surgery and postoperative chemotherapy with gemcitabine and paclitaxel. However, 2 years later, a metastatic lung tumor appeared. Recurrence of the clear cell carcinoma was suspected. The tumor disappeared after 6 courses of gemcitabine and carboplatin, and radiation therapy was performed at the obstetrics and gynecology department. In 2016, follow‐up CT scans showed an increase in the cystic lesions of the left kidney, so the patient was referred to our department.

**Fig. 1:**
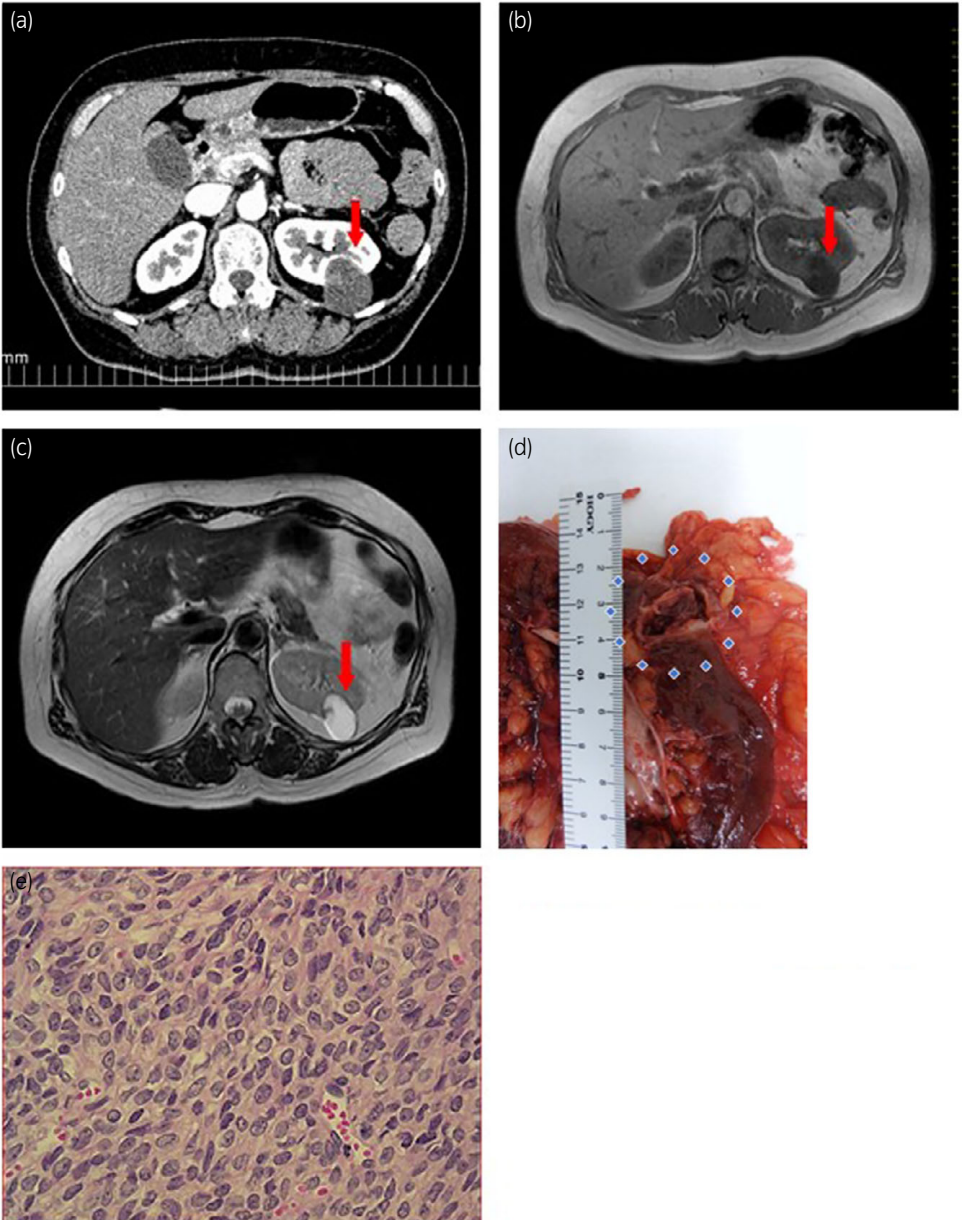
(a) CT scan image. (b) MRI T1 image. (c) MRI T2 image. (d) Macroscopic findings of GCT. (e) Microscopic finding of renal tumor (original magnification ×100).

On CT scans, Heterogeneous partition‐like and solid parts were also observed, which correspond to Bosniak category III (Fig. 1a). MRI T1 displayed a low signal, and T2 displayed a high signal (Fig. 1b,c). Also, some solid components were detected inside. Ultrasound images showed blood flow within the cyst. Based on these results, cystic renal cell carcinoma was suspected, and partial nephrectomy was scheduled. However, while waiting for surgery for over 3 months, the tumor’s diameter increased from 2.5 to 3.9 cm and the solid components became clear. When we explained tumor growth to the patient, she strongly desired to remove her left kidney. Thus, we scheduled a total left nephrectomy.

**Fig. 2:**
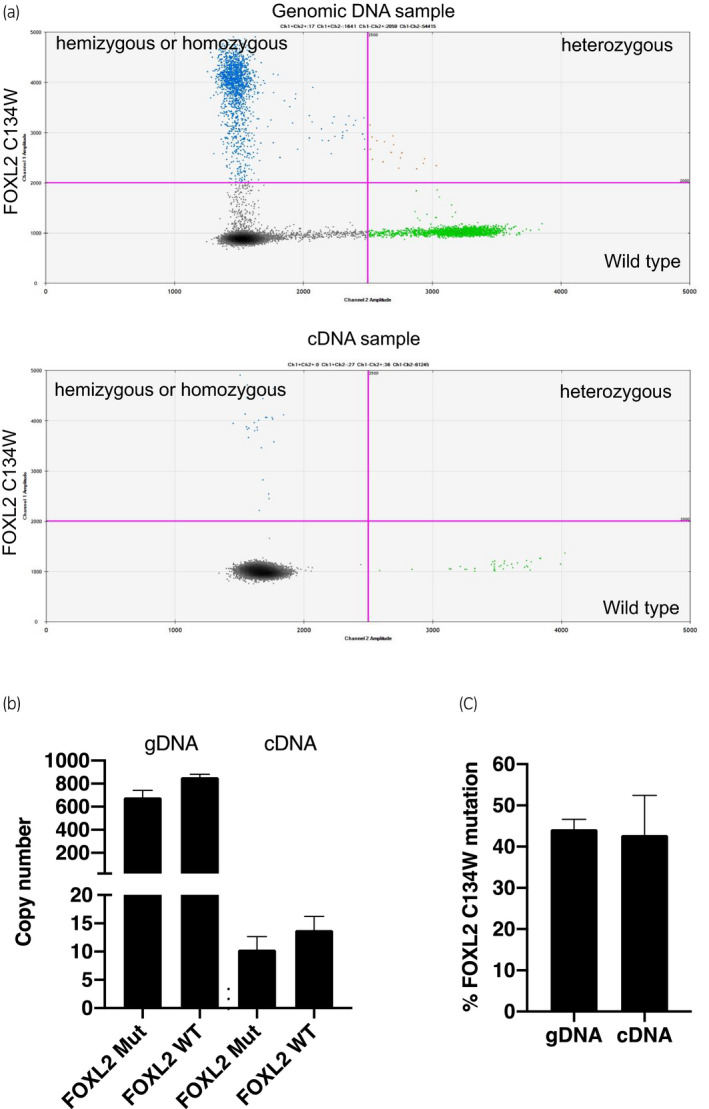
ddPCR results of the *FOXL2* 402C→G missense mutation. (a) Validation of the *FOXL2* 402C→G mutation with the use of a ddPCR assay shows a clear division between samples that were hemizygous or homozygous for the mutation (presumably through chromosome‐based loss of heterozygosity); both homozygous (blue dot) and heterozygous (orange dot) mutations were found in gDNA sample. Only hemizygous or homozygous mutation was found in cDNA samples. (b) Copy number of *FOXL2* 402C→G mutation and *FOXL2* wild type in gDNA and cDNA. Panel 3. Mutation prevalence of *FOXL2* C134W mutation analyzed by ddPCR.

**Fig. 3:**
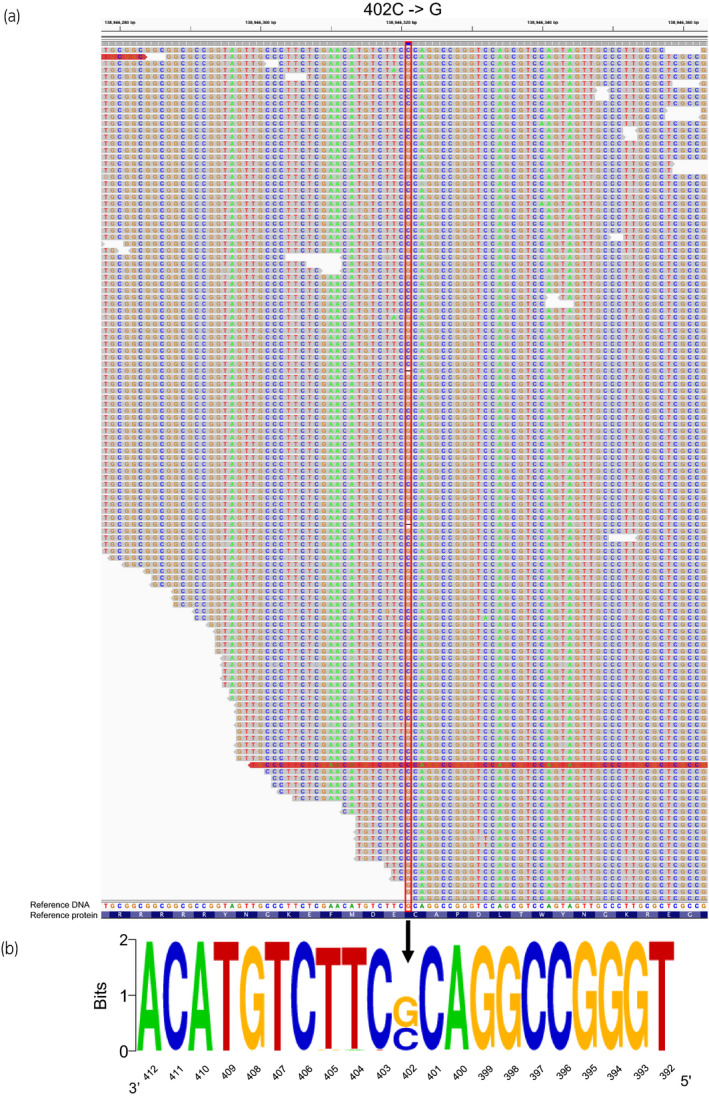
The *FOXL2* 402C→G missense mutation. (a) A whole exome sequence results that the mapped sequence reads from patients on chromosome 3 for genomic positions 138946278 to 138946363 of Human GRch38 (NC.000003.12). cDNA position for *FOXL2* 402 is outlined in red, along with the non‐reference G alleles. Reference gDNA (human Grhg38) and protein sequences with the mutated residues are indicated by red boxes. (b) Sequence logos 21 represent the allele distribution of the position of the mutation and surrounding nucleotides. A measure of 2 bits represents the homozygous position. The variant 402C→G is clearly visible in each logo that was heterozygous for the *FOXL2* mutation.

In 2016, the patient underwent a laparoscopic left nephrectomy. The surgical specimen macroscopically showed the formation of a 3.9‐cm‐diameter cyst with grayish‐white solid lesions (Fig. 1d). The tumor cells found in the solid lesions had constricted nuclear margin, a coffee bean‐like nuclear groove, and mitotic figures (Fig. 1e). Furthermore, immunostaining was positive for SF1 and α‐inhibin. A genetic test using FFPE samples was performed to evaluate the presence of a 402C→G (C134W) mutation of *FOXL2*, which is specific to AGCT, and the test confirmed the presence of the mutation. Total RNA and gDNA were extracted from tumor tissue that was macro‐dissected from the 20 μm‐thickness FFPE tissue section. We conducted mutation analysis of *FOXL2* in FFPE GCT tissues by PCR and whole genome sequence. Per analysis results, *FOXL2* C134W mutation was present in the patient’s sample (Figs 2 and 3).^1^, ^2^, ^3^

The final diagnosis was renal metastasis of the ovarian GCT, and no residual tumor was detected after consultation with a gynecologist. GCTs sometimes cause genital bleeding and dysmenorrhea due to estrogen production. Unfortunately, serum estrogen was not measured before and after surgery, and there were no estrogen‐related clinical symptoms in this patient. Thus, the patient was followed up without additional treatment. There was no recurrence for 5 years after nephrectomy.

## Discussion

Ovarian GCTs originate from follicular granulosa cells and account for 2–5% of all ovarian malignancies whereas AGCTs account for 95% of all GCTs.^4^ Most cases of AGCT are diagnosed at an early stage. First‐line treatment is surgery, and chemotherapy and radiation therapy for recurrence, metastasis, and residual tumors. The 5‐year survival rate is not high (59%) in the third and fourth stages. AGCT recurs in approximately 20–30% of cases, most of which occur later in life.^4^ Recurring AGCTs mainly grow in the pelvis and abdominal cavity, and metastasize to the lungs, liver, and pancreas. Metastases to the kidney are extremely rare, and we found only one case in the literature.^5^ To date, GCTs remain extremely difficult to treat after surgery and during long‐term follow‐up.

In 2009, Shah *et al*. reported that the C134W gene mutation of *FOXL2* is specific to AGCT.^1^ *FOXL2* is a member of the forkhead–winged‐helix family of transcription factors. *FOXL2*, a gene identified in patients with primary ovarian insufficiency, is predominantly expressed in granulosa cells and contributes to the production of estrogen and progesterone and follicle development.^1^ *FOXL2* continuously suppresses SRY's target gene *Sox9* (inducing undifferentiated cells, sperm cells, and Sertoli cells).^6^ In mice with deactivated *FOXL2*, an increase in *Sox9* was observed with or without SRY, and it was found that granulosa cells and follicular cells were reconstructed into testis‐like cells and the ovary.^6^ *FOXL2* 402C→G mutation is present in approximately 95% of AGCTs,^2^ and this mutation is present in the patient’s renal metastasis specimen.

## Conclusion

We report a very rare case of renal metastasis of an ovarian GCT with *FOXL2* mutation 14 years after initial treatment. There was no recurrence 5 years after the left nephrectomy, but close monitoring during follow‐up is needed for a possible late recurrence.

## Author contributions

Kyo Togashi: Conceptualization; Writing – original draft; Writing – review & editing. Tohru Yoneyama: Data curation; Investigation. MIhoko Sutoh Yoneyama: Data curation; Investigation. Hayato Yamamoto: Data curation. Shingo Hatakeyama: Data curation. Takahiro Yoneyama: Data curation. Yasuhiro Hashimoto: Data curation; Writing – review & editing. Masayuki Futagami: Data curation. Chikara Ohyama: Supervision.

## Conflict of interest

The authors declare no conflict of interest.

## Approval of the research protocol by an Institutional Reviewer Board

The protocol for this research project has been approved by a suitably constituted Ethics Committee of our institution.

## Informed consent

Informed consent was obtained from the subjects.

## Registry and the Registration No. of the study/trial

Not applicable.

## References

[1] Shah SP , Kobel M , Senz J *et al*. Mutation of FOXL2 in granulosa‐cell tumors of the ovary. N. Engl. J. Med. 2009; 360: 2719–29. 19516027 10.1056/NEJMoa0902542

[2] Groeneweg JW , Roze JF , Peters EDJ *et al*. FOXL2 and TERT promoter mutation detection in circulating tumor DNA of adult granulosa cell tumors as biomarker for disease monitoring. Gynecol. Oncol. 2021; 162: 413–20. 34083028 10.1016/j.ygyno.2021.05.027

[3] Jamieson S , Butzow R , Andersson N *et al*. The FOXL2 C134W mutation is characteristic of adult granulosa cell tumors of the ovary. Mod. Pathol. 2010; 23: 1477–85. 20693978 10.1038/modpathol.2010.145

[4] Hillman RT , Lin DI , Lawson B , Gershenson DM . Prevalence of predictive biomarkers in a large cohort of molecularly defined adult‐type ovarian granulosa cell tumors. Gynecol. Oncol. 2021; 10.1016/j.ygyno.2021.06.024. PMC9448642 34238613

[5] Burns EM , Rosoff JS , Brooks SA , Picard MM , Smith MT , Picard JC . Renal metastasis of an ovarian granulosa cell tumour inducing growth of a cystic nephroma. BMJ Case Rep. 2013; 2013. 10.1136/bcr-2013-200010. PMC3736646 23843415

[6] Uhlenhaut NH , Jakob S , Anlag K *et al*. Somatic sex reprogramming of adult ovaries to testes by FOXL2 ablation. Cell 2009; 139: 1130–42. 20005806 10.1016/j.cell.2009.11.021

